# Modeling colorectal cancer evolution

**DOI:** 10.1038/s10038-021-00930-0

**Published:** 2021-05-13

**Authors:** Atsushi Niida, Koshi Mimori, Tatsuhiro Shibata, Satoru Miyano

**Affiliations:** 1 Laboratory of Molecular Medicine, Human Genome Center, The Institute of Medical Science, The University of Tokyo, Tokyo, Japan; 2 Department of Surgery, Kyushu University Beppu Hospital, Beppu, Japan; 3 Division of Cancer Genomics, National Cancer Center Research Institute, Tokyo, Japan; 4 M&D Data Science Center, Tokyo Medical and Dental University, Tokyo, Japan

**Keywords:** Cancer genomics, Computational biology and bioinformatics

## Abstract

Understanding cancer evolution provides a clue to tackle therapeutic difficulties in colorectal cancer. In this review, together with related works, we will introduce a series of our studies, in which we constructed an evolutionary model of colorectal cancer by combining genomic analysis and mathematical modeling. In our model, multiple subclones were generated by driver mutation acquisition and subsequent clonal expansion in early-stage tumors. Among the subclones, the one obtaining driver copy number alterations is endowed with malignant potentials to constitute a late-stage tumor in which extensive intratumor heterogeneity is generated by the accumulation of neutral mutations. We will also discuss how to translate our understanding of cancer evolution to a solution to the problem related to therapeutic resistance: mathematical modeling suggests that relapse caused by acquired resistance could be suppressed by utilizing clonal competition between sensitive and resistant clones. Considering the current rate of technological development, modeling cancer evolution by combining genomic analysis and mathematical modeling will be an increasingly important approach for understanding and overcoming cancer.

## Introduction

Colorectal cancer is one of the most prevalent and deadly tumor types in both men and women worldwide. Patients are often diagnosed at an advanced stage, when tumor cell dissemination has taken place, and provided with a limited increase in overall survival by chemo- and targeted therapies due to therapeutic resistance. Although not limited to colorectal cancer, a clue to overcome the therapeutic difficulty resides in understanding the evolution of cancer cells. During tumorigenesis, normal cells are transformed into malignant cancer cells through the accumulation of mutations and natural selection. Evolution allows cancer cells to adapt to new environments and to acquire malignant phenotypes, such as metastatic potential and resistance to treatment. The idea that cancer is an evolutionary system was first proposed by Nowell in 1976 [1]. Subsequent discoveries of oncogenes and tumor suppressor genes (collectively referred to as driver genes) were integrated into this view, leading to Fearon and Vogelstein’s multistep carcinogenesis hypothesis [2]: in colorectal tumorigenesis, while accumulating multiple driver genes including *APC*, *KRAS*, *TP53*, and *SMAD4*, a normal epithelial cell linearly transforms through a benign lesion into a malignant tumor. Since then, tumorigenesis has been viewed as a linear evolutionary process of malignant transformation through repeated acquisition of driver mutations and Darwinian selection.

However, since the advent of next-generation sequencing, this view has changed dramatically [3]. In particular, multiregion sequencing, in which we analyze multiple samples obtained from physically separate regions within the tumor of a single patient, has revealed that solid tumors harbor extensive intratumor heterogeneity (ITH) formed by branching evolutionary processes. Through multiregion sequencing, we identified two categories of somatic single-nucleotide mutations: “founder” and “progressor” mutations. Founder mutations are defined to be present in all of the regions while progressor mutations are defined to be present in some of the regions (note that they are also referred to by different terms in different studies, e.g., public/private or trunk/branch mutations). Founder mutations are assumed to accumulate during the early phases of cancer evolution. The common ancestor clone that has acquired all the founder mutations then branches into subclones, which accumulate progressor mutations and contribute to the formation of ITH. Through these multiregion mutational profiles, we can infer an evolutionary history of the cancer by constructing a phylogenetic tree.

For example, whole-exome multiregion sequencing using multiple samples from primary and metastatic lesions in 10 patients with renal cancer revealed extensive ITH and clonal branching evolution [4, 5]. Their study also revealed not only founder non-silent mutations in some known driver genes such as *VHL*, but also progressor non-silent mutations in other known driver genes such as *SETD2* and *BAP1*. It is intriguing that, in some cases, different mutations of the same driver gene or pathway were acquired independently. This phenomenon called parallel evolution also indicates that a part of the ITH was generated by Darwinian selection.

### Neutral evolution in advanced colorectal cancer

**Fig. 1:**
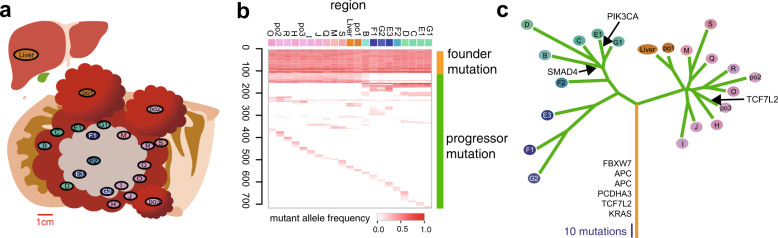
Multiregion sequencing of colorectal cancer. **a** A schema of a multiregion sampling in a primary colorectal cancer and matched metastatic liver lesion. In this case, we obtained 20 samples from the primary lesion and one sample from the metastatic lesion. **b** A multiregion mutation profile. The depth of red represents mutant allele frequency while the colors of sample labels were prepared so that the similarities of colors represent those of mutation patterns. **c** A phylogenetic tree constructed from the multiregion mutation profile. The time when mutations in known driver genes of colorectal cancer are acquired is indicated along the tree. This image was obtained by modifying a figure which originally appeared in our previous work [6]

Inspired by this pioneering study, we investigated ITH in nine cases of surgically resected late-stage colorectal tumors through whole-exome multiregion sequencing to identify founder and progressor mutations in each case [6]. The results obtained from one of the nine cases are shown in Fig. 1. Progressor mutations showed a mutational pattern that was geographically correlated with the sampling locations. Moreover, we found that, in each region, founder mutations existed as clonal mutations, while progressor mutations existed as subclonal mutations. This finding suggests that, even in each region, extensive ITH existed, which was not captured by the resolution of multiregion sequencing. In addition, most of the mutations in known driver genes such as *APC* and *KRAS* were found to be founder mutations. However, progressor mutations containing few driver mutations and parallel evolution were not confirmed, which is in contrast to the findings obtained in renal cancer. These observations suggest that apart from Darwinian selection, there are other evolutionary principles generating ITH.

In pursuit of these principles, we employed mathematical modeling of the evolutionary process generating ITH using an agent-based model [7]. The agent-based model assumes a set of system constituents, called independent agents, and specifies rules for the independent behavior of the agents themselves, as well as for the interaction between agents and the agent environment. The agent-based model is a flexible representation of the model, and given the initial conditions and parameters of the system, the behavior of the system can be easily analyzed by computational simulation. In the case of modeling the evolution of cancer, if each cell is assumed to be an agent, ITH can be easily represented by the differences in the internal states of each agent. For example, in a pioneering model, agents were assumed to be cells that contained a few genes and proliferated while accumulating mutations. As a result, computer simulation succeeded in reproducing ITH observed in single-gene-focused experiments [8]. Since then, multiple mathematical modeling studies employing agent-based models have been developed to shed light on the principles underlying the generation of ITH. For example, stem cell hierarchy may contribute to ITH, and the interaction between cells as well as the turnover of cells in three-dimensional space may affect the formation of ITH [9].

**Fig. 2:**
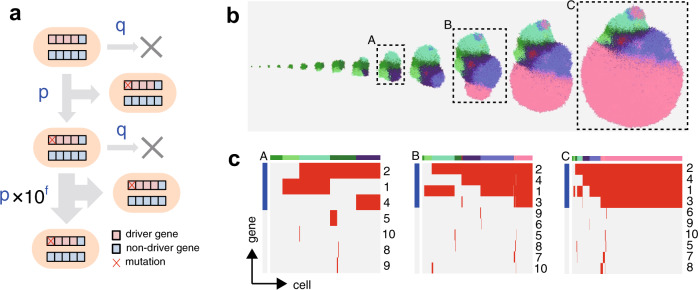
Branching evolutionary process (BEP) model. **a** Each cell has *n* genes (10 genes in this toy model) while each cell divides and dies in a unit time with a probability *p* and *q*, respectively. When the cell divides, each gene is mutated with probability *r*. If any of *d* driver genes (four genes in this toy model) are mutated, the division probability *p* increases 10^*f*^-fold per mutation. **b** Evolutionary snapshots obtained by simulating 2-D tumor growth based on the BEP model with an appropriate parameter setting. The region with the same color represents a clone with the same set of mutated genes. **c** Single-cell mutation profiles at three time points in the simulated tumor growth. Top colored bands represent clones, while the blue bands on the left represent driver genes. This figure was obtained by modifying a figure which originally appeared in our previous work [53]

Because the existing models could not completely reproduce the extensive ITH revealed by our multiregion sequencing of colorectal cancer, we developed a new agent-based model, the branching evolutionary process (BEP) model, to simulate heterogeneous cancer evolution [6]. Similar to the other models, the BEP model assumes cells to be agents (Fig. 2a). Each cell harbors *n* genes, including *d* driver genes, while each cell divides and dies in a unit time with a probability *p* and *q*, respectively. When the cell divides, each gene is mutated with probability *r*, and if any driver genes are mutated, the division probability *p* increases by 10^*f*^-fold per mutation. In the BEP model, *f* can be regarded as the strength of driver genes. Given that a cell without mutations divides according to this rule, after the normal cell acquires the first driver mutation, which accelerates cell division, the proportion of the clone originating from the cell increases in a whole cell population. By repeating these steps, each cell gradually accumulates driver mutations as well as accompanying passenger mutations, which do not affect the cell division rate, and finally, a tumor is formed with numerous mutations accumulated. Depending on the parameter values during the course of cancer evolution, each cancer cell can accumulate different combinations of mutations to generate different types of ITH. In Fig. 2b, c, an example of snapshots of two-dimensional tumor growth is shown, which was simulated using the BEP model with appropriate parameter values. In this example, driver mutations gradually accumulated in the cells, and a clone with four mutations was selected through Darwinian selection, which finally became dominant in the tumor.

**Fig. 3:**
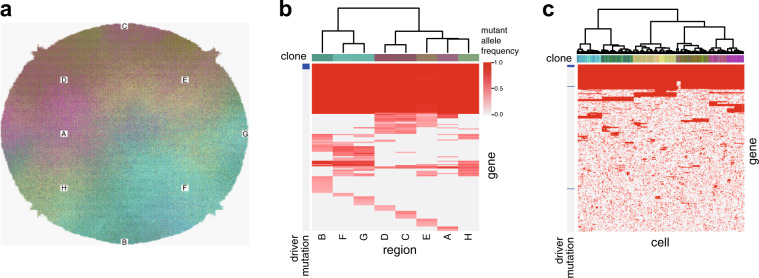
A computer-simulated tumor with extensive intratumor heterogeneity (ITH) generated by neutral evolution. **a** A tumor depicted by branching evolutionary process (BEP) simulation with an assumption of a high mutation rate. **b** A simulated multiregion mutation profile of the simulated tumor. Cell populations in the regions labeled with A-H (**a**) were extracted and their averaged mutation profiles were obtained. Note that the simulated profile is similar to the real one obtained from the colorectal cancer (Fig. 1b), and that driver mutations consisted only of founder mutations. **c** A simulated single-cell mutation profile of the simulated tumor, suggesting the existence of numerous clones that cannot be detected by multiregion sequencing. This image was obtained by modifying a figure which originally appeared in our previous work [6]

To find the principle generating ITH, we performed a large number of BEP simulations using a supercomputer with various parameter settings to find conditions leading to the extensive ITH observed in our genomic analyses [6]. As a result, when cancer evolution was simulated with the assumption of a high mutation rate, followed by computer simulation of multiregion sequencing, we could reproduce mutation profiles similar to those obtained by our multiregion sequencing of colorectal cancers (Fig. 3a, b). That is, irrespective of the presence of founder mutations, progressor mutations contributed to the formation of a heterogeneous mutation profile, which was geographically correlated with sampling locations. Moreover, we could also reconstruct local heterogeneity, as illustrated by the finding that progressor mutations existed as subclonal mutations in each region. Intriguingly, while driver mutations were acquired as founder mutations, progressor mutations contained few driver mutations, and most of them comprised neutral mutations that did not affect the cell division rate. This suggests that, after the appearance of the common ancestor clone with accumulated driver mutations, extensive ITH was generated by neutral evolution. Moreover, single-cell mutation profiles of the simulated tumor suggest that the tumor comprises a large number of minute clones with numerous neutral mutations accumulated (Fig. 3c).

Similar to our study, other studies employed multiregion genomic analysis and mathematical modeling to propose a neutral evolution model to explain the origin of ITH in colorectal tumors [10] and liver cancer [11]. In contrast, an increasing number of multiregion genomic studies have demonstrated different evolutionary properties of ITH among cancer types: non-neutral, neutral, and in-between. [12]. It should also be noted that although we mentioned earlier that the signature of Darwinian selection (i.e., the presence of subclonal driver mutation) is prominent in renal cancer, a large-scale multiregion cancer genome project, TRACERx Renal, identified the renal cancer subtype without the signature of Darwinian selection [13]. The clinical significance of different ITH properties remains unclear but has been proposed to be associated with clinical disease course [10]. A few studies employing mathematical modeling have also been performed to understand the mechanisms generating ITHs with different evolutionary properties [14, 15].

### Evolutionary shift during colorectal tumorigenesis

**Fig. 4:**
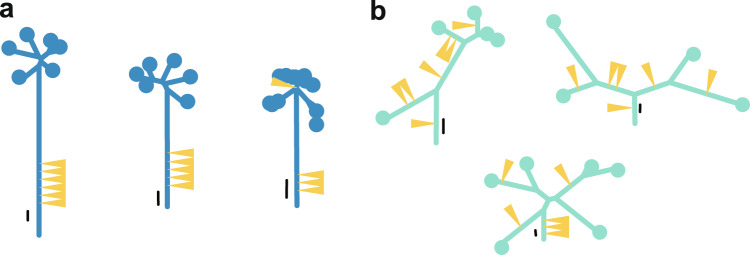
Typical representatives of phylogenetic trees of colorectal cancers. **a** Trees of late-stage tumors, which show “palm tree-like” shapes. **b** Trees of early-stage tumors, which shows “forked tree-like” shapes. Yellow triangles denote driver mutations while black lines near the roots of the trees represent scales for 10 mutations. This image was obtained by modifying a figure which originally appeared in our previous work [16]

As described so far, by combining genomic analysis and mathematical modeling, we demonstrated that neutral evolution shapes the ITH of advanced colorectal cancer. The next question then arises: When is the ITH generated? To answer this question, we performed multiregion sequencing of nine early-stage colorectal tumors, which were resected through endomicroscopic surgery [16]. As a result, we found that extensive ITH was already generated in the early stages of colorectal tumorigenesis. However, compared with late-stage tumors, early-stage tumors demonstrated the enrichment of driver genes in progressor mutations, suggesting the contribution of Darwinian selection. The difference in evolutionary modes is evident in the shapes of phylogenetic trees in which the lengths of the trunk and branches represent the number of founder and progressor mutations, respectively (Fig. 4). The late-stage tumors tended to have “palm tree-like” shapes that were composed of long trunks and short branches. In contrast, the early-stage tumors tended to have “forked tree-like” shapes that were composed of short trunks and long branches. It has been reported that the contribution of Darwinian selection to ITH can also be measured by the distribution of mutant allele frequencies (MAFs) in single-region sequencing data [17]; if a set of subclonal mutations comprised driver and accompanying passenger mutations, Darwinian selection should have made their MAFs high, compared with those from a set without driver mutations. By applying this idea to multiregion analysis, we examined MAFs in each sample of multiregion data to find that MAFs of progressor mutations, especially mutations shared by multiple samples, tended to shift to a higher level in early-stage tumors than in late-stage tumors. Collectively, these results demonstrate that Darwinian selection contributes to the formation of ITH in the early stages of colorectal tumorigenesis. It should be noted that, consistently to our data, another study has independently reported that early-stage colorectal tumors harbor subclonal driver mutations [18].

Although ITH exists in both early-stage and late-stage colorectal tumors, the evolutionary principle generating ITH shifts from Darwinian selection to neutral evolution. Next, we had to understand the mechanism underlying the temporal shift. To solve this problem, we focused on copy number alterations (CNAs). By comparing copy number profiles inferred from exome sequencing data, we found that CNAs drastically increased during the progression from the early stage to the late stage of colorectal tumorigenesis [16]. Notably, although some of the early-stage tumors that we analyzed contained not only adenoma but also carcinoma samples, we observed a significant increase in CNAs in the carcinoma samples alone. In addition to single-nucleotide mutations that we discussed so far, recent studies have demonstrated that, in multiple types of cancers, more drastic chromosome- and/or genome-wide evolutionary events that produce CNAs and chromosomal rearrangements may have occurred in a short time at the early stage of cancer evolution [19, 20]. Such large-scale events could confer a marked fitness increase on one or a few cells, which expand to constitute the tumor mass uniformly. This type of evolution is referred to as “punctuated evolution” after the term “punctuated equilibrium,” which was proposed for species evolution by Gould and Eldredge to challenge the long-standing paradigm of gradual Darwinian evolution [21] although the underlying molecular mechanisms that cause rapid bursts of change are very different. Based on our observation of the drastic increase in CNAs, we hypothesized that punctuated evolution triggers the temporal shift of the evolutionary principle generating ITH.

**Fig. 5:**
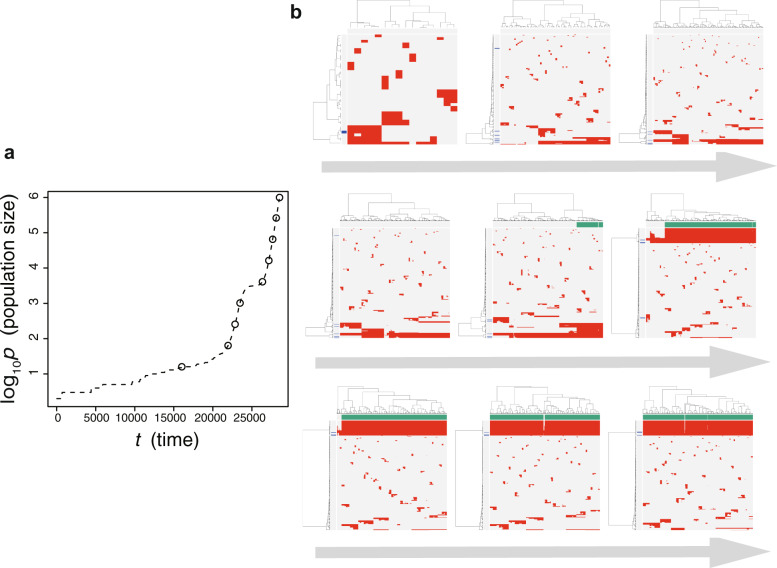
Simulation of the evolutionary shift based on the modified BEP model. **a** growth curve of a simulated tumor. Note that, as the population size *p* approaches 10^5^, which is the carrying capacity value in this example, the growth speed slows down. **b** Evolutionary snapshots of single-cell mutation profiles along the simulated tumor growth. The time points when the snapshots were obtained are indicated by empty circles on the growth curves. Rows and columns of the clustered single-cell mutations profile matrices denote mutations and cells, respectively. Top green bands represent a clone acquiring an explosive driver mutation, while the blue bands on the left represent driver mutations. Note that the driver mutation exists as subclonal mutations at the beginning but shifts to clonal mutations, as the clone acquiring the explosive driver mutation expands. This image was obtained by modifying a figure which originally appeared in our previous work [14]

To examine this hypothesis, we employed mathematical modeling [14]. We modified the BEP model to reproduce punctuated evolution. In the original BEP model, we assumed that a cell can grow infinitely without a decrease in their growth speed. However, it is more natural to assume that there exists a limit of population size because of resource limitations and that the growth speed gradually slows down as the population size approaches the limit. The limit in the population size is called the carrying capacity and is employed in the well-known logistic equation [22]. By mimicking the logistic equation, we introduced the carrying capacity into the modified model and additionally employ an “explosive” driver mutation, which negates the effect of the carrying capacity; that is, it is assumed that the explosive driver mutation rapidly evolves the cell such that it can conquer the growth limit and attain infinite proliferation ability. We show the results of a simulation based on the modified model in Fig. 5. It was observed that multiple subclones with different driver genes coexist; that is, ITH shaped by Darwinian selection is prominent during the early phase of the simulation. Note that a growth curve plot indicates that, as the population size approaches the carrying capacity, the growth speed slows down; however, the tumor regrows after the appearance of a clone that has acquired an explosive driver mutation. Darwinian selection makes the clone with the explosive driver mutation dominant in the population, which causes subclonal driver mutations in the clone to shift to clonal mutations. Then, neutral mutations alone accumulate as subclonal mutations; that is, ITH is finally generated by neutral evolution. Collectively, our mathematical modeling also supports the notion that punctuated evolution triggers an evolutionary shift in colorectal tumorigenesis.

**Fig. 6:**
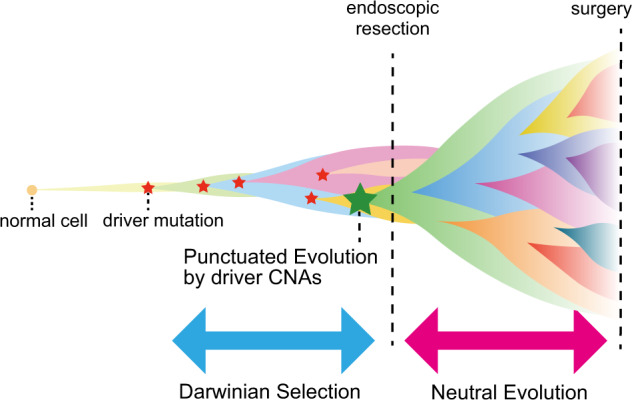
Our model of colorectal cancer evolution. During early tumorigenesis, multiple subclones harboring different single-nucleotide mutations appear and constitute ITH by Darwinian selection. The tumor is then confronted with growth limitation before progressing to the late phase of tumorigenesis. Out of the multiple subclones generated by Darwinian selection, the parental clone that can conquer the growth limitation emerges. In addition to a sufficient set of driver mutations, such a clone acquires driver CNAs. The parental clone is selected to progress locally advanced cancer or metastatic cancer. During the late phase, extensive ITH is generated by neutral evolution. This image was obtained by modifying a figure which originally appeared in our previous work [16]

In summary, our genomic analysis and mathematical modeling led us to develop a new model of colorectal cancer evolution (Fig. 6). In our model, multiple subclones are generated by driver mutation acquisition and subsequent clonal expansion in an early-stage tumor; however, early tumor growth is inevitably hampered by obstacles such as spatial and nutritional limitations and immune attack. However, of the multiple subclones generated by Darwinian selection, a parental clone that can overcome the obstacles emerges. In addition to a sufficient set of driver mutations, such a clone acquires driver CNAs that endow a tumor with malignant phenotypes, such as invasion, angiogenesis, and immune escape, and then it dominantly regrows by overcoming the obstacles. Reinforcing this view, we recently disclosed that the arm-level CNA in cancer tissues elicits immune tolerance in the cancer microenvironment, such as impaired cytolytic activity and diminished expression of cytotoxic cell-related genes [23]. After punctuated evolution, numerous subclones were generated by the accumulation of neutral mutations.

Our model is consistent with the well-established multi-step carcinogenesis model of CRC [2], in which mutations in major driver genes such as APC, KRAS, and TP53 are sequentially accumulated in adenoma and then additional CNAs are acquired during the progression from adenoma to carcinoma. The neutral evolution phase following punctuated evolution is also consistent with the recently proposed Big Bang model [10], where a tumor predominantly grows as a single expansion without selective sweep. It should also be noted that our model of the evolutionary shift from Darwinian selection to neutral evolution might be a simplified view; different evolutionary processes actually work not separately but simultaneously and continuously as a series of phases of cancer evolution, which is discussed in our previous paper [14].

Either way, our evolution model is far from complete, and many aspects remain to be done. Our model is a rough sketch of the evolution of microsatellite stable tumors, which is the major subtype of colorectal cancer. Since the minor subtype, microsatellite instable tumors, generally shows higher mutation rates and lower CNA rates than microsatellite stable tumors, our evolution model is not applicable [24]. Subtypes exist even in microsatellite stable tumors. For example, we identified depressed-type carcinoma, which is characterized by a depressed surface in colorectal mucosa, as early-stage lesions for a possible novel subtype of a microsatellite stable tumor. The depressed cancers were positively correlated with lymphovascular invasion, tumor budding, and massive submucosal invasion. Our genomic analysis also demonstrated that depressed carcinomas harbor arm-level copy number amplification in 13q and 20p more frequently than protruding carcinomas [25]. The construction of a subtype-specific evolution model should be addressed in future studies.

Although clinically important, the evolutionary path to metastasis was not addressed in our model. Recently, several studies employing multiregion sequencing have successfully uncovered metastatic routes in colorectal cancer [26–28]. So far, no driver events that specifically occurred in metastatic samples have been identified, suggesting that metastatic potential is already acquired in founder clones, consistent with our neutral evolution model. Mathematical modeling of the multiregion data indicated that metastatic clones tended to branch out from the primary tumor in the early phase of evolution, which is in contrast with the case presented in Fig. 1 [29]. Another study employing mathematical modeling proposed that fewer primary tumor lineages seed distant metastases than lymph node metastases, that is, different levels of selection work against the two sites [30]. The evolution and ITH of tumor-immune interactions should also be studied in greater depth.

Recently, studies that integrated multiregion immunogenomic data such as human leukocyte antigens, neoantigens, T cell receptor repertoire, and expression of immune-related genes have been reported for several cancer types [31–34]. Although much work on colorectal cancer remains to be done, such an approach might provide insight into the mechanism underlying resistance against immune checkpoint inhibitors, which is only approved and partially effective for microsatellite instable tumors [32].

### Evolution-based strategy for coping with therapeutic resistance

Finally, we discuss on how to utilize our understanding of cancer evolution to address the problems related to therapeutic resistance. Currently, a large number of molecular target drugs are available or under development. Targeting driver genes in colorectal cancer appears to be a rational approach when considering our evolution model where driver genes exist as founder mutations. However, drug resistance frequently appears during therapy for most drugs, leading to therapeutic failure. Our model indicates that late-stage colorectal tumors harbor extensive ITH generated by neutral evolution, which could be a fundamental cause of therapeutic failure. Whether a mutation is neutral or not depends on the surrounding environment, and an environmental change induced by a specific therapy can convert a neutral mutation that has no selective advantage before the therapy to a driver mutation leading to therapeutic refractoriness (resistant mutation). This means that any type of therapy can potentially generate a resistant clone with a resistant mutation, which leads to tumor relapse even if the therapy is temporarily effective.

**Fig. 7:**
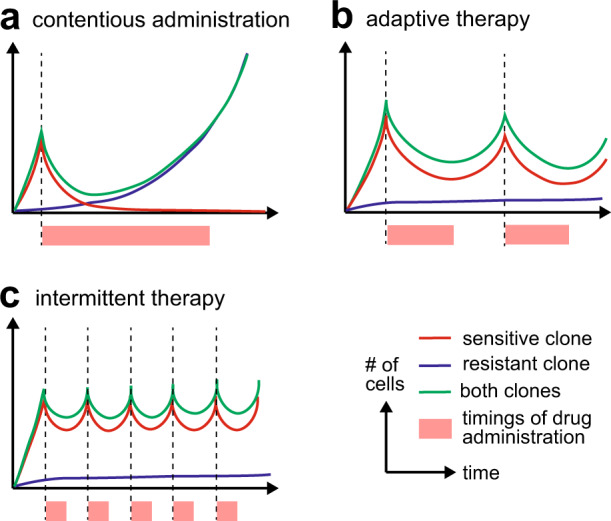
Evolution-based therapeutic strategy. **a** Under contentious administration, the tumor temporarily shrinks but the relapse is inevitable due to the expansion of a resistant clone. **b, c** It is theoretically possible that adaptive therapy (**b**) or intermittent therapy (**c**) suppress the relapse utilizing clonal competition

Studies employing mathematical modeling have shown that tumor regrowth can be delayed or prevented by adjusting the therapeutic regimen [35, 36]. In normal clinical practice, an anticancer drug is continuously administered to a patient with cancer at the maximum tolerated dose, if possible. Given that a tumor comprises major and minor clones that are sensitive and resistant to chemotherapy, respectively, the tumor temporarily shr inks since the major drug-sensitive clone is eradicated. However, as the major drug-sensitive clone disappears, the minor resistant clone can grow freely because of the release from growth competition, that is, the competitive relationship between the two clones is dissolved (Fig. 7a). In contrast, if we can keep the two clones in a competitive state while controlling the total tumor volume within an acceptable threshold, the survival of the patient can be prolonged as compared with the routine continuous administration.

For example, in “adaptive therapy,” the initial dose of an anticancer drug is high, and then the dosage is decreased as the tumor shrinks to eventually maintain the sensitive clone at a level sufficient to suppress the growth of the resistant clone (Fig. 7b) [37]. In fact, in an experimental system using xenografts, it has been shown to prolong the survival rate of mice compared with the standard dosing schedule [38]. In antiandrogen therapy for prostate cancer, continuous administration causes adverse events, leading to poor quality of life. To address this problem, “intermittent therapy” (Fig. 7c) has been proposed [39] in which administration is repeated cyclically while monitoring the level of serum prostate-specific antigen (PSA), a noninvasive biomarker quantifying prostate tumor growth. Current data do not show that intermittent therapy is inferior to continuous therapy, with statistical certainty. Moreover, the rationality of intermittent therapy is provided by the mathematical modeling of tumor growth dynamics measured by PSA [40, 41]; that is, intermittent therapy can suppress relapse by utilizing clonal competition, similarly to adaptive therapy.

Although the availability of noninvasive biomarkers such as PSA is essential for mathematical modeling of actual clinical data, recent advances in liquid biopsy technology have made it possible to take a similar approach for other cancer types. In particular, liquid biopsy based on circulating tumor DNA (ctDNA) appears to be a promising tool for this purpose [42]. ctDNA is a tumor-derived portion of cell-free DNA (cfDNA), which is all non-encapsulated DNA circulating in the bloodstream. By applying digital PCR or deep sequencing to cfDNA extracted from patients’ plasma, we can non-invasively detect mutations in ctDNA. The allele frequencies of the mutations in ctDNA are supposed to reflect the real-time clonal proportions in the whole tumor, including primary and metastatic lesions, which can provide an opportunity for tracking clonal dynamics during a therapeutic course.

A few pioneering studies have recently combined ctDNA-based liquid biopsy with mathematical modeling to understand the therapeutic resistance in colorectal cancer. For example, the time-series data acquired by digital PCR of cfDNA showed the emergence of a resistant *KRAS* mutation during anti-*EGFR* therapy in patients with metastatic colorectal cancer. Moreover, mathematical analysis of the time-series data suggested that the *KRAS* mutation already existed in the tumor before the initiation of chemotherapy, which is consistent with the view derived from our neutral evolution model [43]. Targeted cfDNA sequencing demonstrated that acquired resistance to anti-*EGFR* therapy results from multiple resistant clones, and mathematical modeling combined with frequent serial sampling of cfDNA allows prediction of the expected time to treatment failure in individual patients [44]. It has also been reported that after discontinuation of anti-*EGFR* therapy, resistant clones decay due to a lack of growth advantage relative to sensitive clones, which supports anti-*EGFR* rechallenge [45]. Collectively, we expect that mathematical modeling of tumor growth data profiled by liquid biopsy will not only help us understand colorectal tumor evolution during anticancer drug therapy but also work out therapeutic strategies for coping with therapeutic resistance.

## Conclusion

In this review, we introduced our works in which we modeled colorectal cancer evolution by genomic analysis and mathematical modeling. The explosion of cancer genomic data still continues on; moreover, technological innovation represented by single-cell sequencing technologies is also accelerating [46]. Although most of the current single-cell sequencing technologies focus on transcriptome analysis, it has been reported that ITH of CNAs can be computationally inferred form single-cell RNA sequencing data　[47]. Recently, a protocol for performing both transcriptomic profiling and targeted mutation detection simultaneously at the single-cell level has also developed [48]. These approaches appear to be useful to study not only genomic evolution itself but also phenotypic changes accompanying it. The resolution of spatial sequencing is approaching to the single-cell level [49]; the spatial sequencing approach will expectedly be a powerful tool for understanding ITH of solid tumors at the ultimate level.

Mathematical modeling incorporating large amount and complexity of data will be empowered by approximate Bayesian computation (ABC) [50]. ABC is a computational method that estimated the parameter distributions of a simulation model. Although ABC is originally introduced in population genetics, it has also recently gained popularity in other fields of biological science. In fact, our work [6] and another work cited above [29] employed ABC for fitting simulation models to real data. Although the performance of ABC depends on the choice of summary statistic, which is used for evaluating similarities between real and simulation data, it has recently been reported that the summary statistic can automatedly be selected by deep learning [51, 52]. It is expected that, together with such methodological improvements, the expansion of computational resources will broaden the applicability of ABC. In summary, considering these recent technological advancements, modeling cancer evolution by combining genomic analysis and mathematical modeling will be an increasingly important approach for understanding and overcoming not only colorectal cancer but also other types of cancer.

## References

[1] Nowell PC (1976). The clonal evolution of tumor cell populations. Science.

[2] Fearon ER, Vogelstein B (1990). A genetic model for colorectal tumorigenesis. Cell.

[3] McGranahan N, Swanton C (2017). Clonal heterogeneity and tumor evolution: past, present, and the future. Cell.

[4] Gerlinger M, Rowan AJ, Horswell S, Math M, Larkin J, Endesfelder D (2012). Intratumor heterogeneity and branched evolution revealed by multiregion sequencing. N Engl J Med.

[5] Gerlinger M, Horswell S, Larkin J, Rowan AJ, Salm MP, Varela I (2014). Genomic architecture and evolution of clear cell renal cell carcinomas defined by multiregion sequencing. Nat Genet.

[6] Uchi R, Takahashi Y, Niida A, Shimamura T, Hirata H, Sugimachi K (2016). Integrated multiregional analysis proposing a new model of colorectal cancer evolution. PLoS Genet.

[7] Macal CM, North MJ, editors. Tutorial on agent-based modeling and simulation. Proceedings of the Winter Simulation Conference, 2005; 2005 4-4 Dec. 2005.

[8] Gonzalez-Garcia I, Sole RV, Costa J (2002). Metapopulation dynamics and spatial heterogeneity in cancer. Proc Natl Acad Sci USA.

[9] Sottoriva A, Verhoeff JJ, Borovski T, McWeeney SK, Naumov L, Medema JP (2010). Cancer stem cell tumor model reveals invasive morphology and increased phenotypical heterogeneity. Cancer Res.

[10] Sottoriva A, Kang H, Ma Z, Graham TA, Salomon MP, Zhao J (2015). A Big Bang model of human colorectal tumor growth. Nat Genet.

[11] Ling S, Hu Z, Yang Z, Yang F, Li Y, Lin P (2015). Extremely high genetic diversity in a single tumor points to prevalence of non-Darwinian cell evolution. Proc Natl Acad Sci USA.

[12] Iacobuzio-Donahue CA, Litchfield K, Swanton C (2020). Intratumor heterogeneity reflects clinical disease course. Nat Cancer.

[13] Turajlic S, Xu H, Litchfield K, Rowan A, Horswell S, Chambers T (2018). Deterministic evolutionary trajectories influence primary tumor growth: TRACERx renal. Cell.

[14] Niida A, Hasegawa T, Innan H, Shibata T, Mimori K, Miyano S (2020). A unified simulation model for understanding the diversity of cancer evolution. PeerJ.

[15] Edwards J, Marusyk A, Basanta D (2021). Selection-driven tumor evolution with public goods leads to patterns of clonal expansion consistent with neutral growth. iScience.

[16] Saito T, Niida A, Uchi R, Hirata H, Komatsu H, Sakimura S (2018). A temporal shift of the evolutionary principle shaping intratumor heterogeneity in colorectal cancer. Nat Commun.

[17] Williams MJ, Werner B, Barnes CP, Graham TA, Sottoriva A (2016). Identification of neutral tumor evolution across cancer types. Nat Genet.

[18] Cross W, Kovac M, Mustonen V, Temko D, Davis H, Baker AM (2018). The evolutionary landscape of colorectal tumorigenesis. Nat Ecol Evol.

[19] Baca SC, Prandi D, Lawrence MS, Mosquera JM, Romanel A, Drier Y (2013). Punctuated evolution of prostate cancer genomes. Cell.

[20] Gao R, Davis A, McDonald TO, Sei E, Shi X, Wang Y (2016). Punctuated copy number evolution and clonal stasis in triple-negative breast cancer. Nat Genet.

[21] Gould SJ, Eldredge N (1993). Punctuated equilibrium comes of age. Nature.

[22] Verhulst PF (1838). Notice sur la loi que la population suit dans son accroissement. Corresp Math Phys.

[23] Sakimura S, Nagayama S, Fukunaga M, Hu Q, Kitagawa A, Kobayashi Y (2021). Impaired tumor immune response in metastatic tumors is a selective pressure for neutral evolution in CRC cases. PLoS Genet.

[24] Boland CR, Goel A (2010). Microsatellite instability in colorectal cancer. Gastroenterology.

[25] Kudo SE, Kouyama Y, Ogawa Y, Ichimasa K, Hamada T, Kato K (2020). Depressed colorectal cancer: a new paradigm in early colorectal cancer. Clin Transl Gastroenterol.

[26] Naxerova K, Reiter JG, Brachtel E, Lennerz JK, van de Wetering M, Rowan A (2017). Origins of lymphatic and distant metastases in human colorectal cancer. Science.

[27] Zhang C, Zhang L, Xu T, Xue R, Yu L, Zhu Y (2020). Mapping the spreading routes of lymphatic metastases in human colorectal cancer. Nat Commun.

[28] Dang HX, Krasnick BA, White BS, Grossman JG, Strand MS, Zhang J (2020). The clonal evolution of metastatic colorectal cancer. Sci Adv.

[29] Hu Z, Ding J, Ma Z, Sun R, Seoane JA, Scott Shaffer J (2019). Quantitative evidence for early metastatic seeding in colorectal cancer. Nat Genet.

[30] Reiter JG, Hung WT, Lee IH, Nagpal S, Giunta P, Degner S (2020). Lymph node metastases develop through a wider evolutionary bottleneck than distant metastases. Nat Genet.

[31] Joshi K, de Massy MR, Ismail M, Reading JL, Uddin I, Woolston A (2019). Spatial heterogeneity of the T cell receptor repertoire reflects the mutational landscape in lung cancer. Nat Med.

[32] McGranahan N, Rosenthal R, Hiley CT, Rowan AJ, Watkins TBK, Wilson GA (2017). Allele-specific HLA loss and immune escape in lung cancer evolution. Cell.

[33] Zhang AW, McPherson A, Milne K, Kroeger DR, Hamilton PT, Miranda A (2018). Interfaces of malignant and immunologic clonal dynamics in ovarian. Cancer Cell.

[34] Rosenthal R, Cadieux EL, Salgado R, Bakir MA, Moore DA, Hiley CT (2019). Neoantigen-directed immune escape in lung cancer evolution. Nature.

[35] Enriquez-Navas PM, Wojtkowiak JW, Gatenby RA (2015). Application of evolutionary principles to cancer therapy. Cancer Res.

[36] Amirouchene-Angelozzi N, Swanton C, Bardelli A (2017). Tumor evolution as a therapeutic target. Cancer Discov.

[37] Gatenby RA, Silva AS, Gillies RJ, Frieden BR (2009). Adaptive therapy. Cancer Res.

[38] Enriquez-Navas PM, Kam Y, Das T, Hassan S, Silva A, Foroutan P (2016). Exploiting evolutionary principles to prolong tumor control in preclinical models of breast cancer. Sci Transl Med.

[39] Perera M, Roberts MJ, Klotz L, Higano CS, Papa N, Sengupta S (2020). Intermittent versus continuous androgen deprivation therapy for advanced prostate cancer. Nat Rev Urol.

[40] Tanaka G, Hirata Y, Goldenberg SL, Bruchovsky N, Aihara K (2010). Mathematical modelling of prostate cancer growth and its application to hormone therapy. Philos Trans A Math Phys Eng Sci.

[41] Jain HV, Clinton SK, Bhinder A, Friedman A (2011). Mathematical modeling of prostate cancer progression in response to androgen ablation therapy. Proc Natl Acad Sci USA.

[42] Wan JCM, Massie C, Garcia-Corbacho J, Mouliere F, Brenton JD, Caldas C (2017). Liquid biopsies come of age: towards implementation of circulating tumour DNA. Nat Rev Cancer.

[43] Diaz LA, Williams RT, Wu J, Kinde I, Hecht JR, Berlin J (2012). The molecular evolution of acquired resistance to targeted EGFR blockade in colorectal cancers. Nature.

[44] Khan KH, Cunningham D, Werner B, Vlachogiannis G, Spiteri I, Heide T (2018). Longitudinal liquid biopsy and mathematical modeling of clonal evolution forecast time to treatment failure in the PROSPECT-C phase II colorectal cancer clinical trial. Cancer Disco.

[45] Siravegna G, Mussolin B, Buscarino M, Corti G, Cassingena A, Crisafulli G (2015). Clonal evolution and resistance to EGFR blockade in the blood of colorectal cancer patients. Nat Med.

[46] Ren X, Kang B, Zhang Z (2018). Understanding tumor ecosystems by single-cell sequencing: promises and limitations. Genome Biol.

[47] Gao R, Bai S, Henderson YC, Lin Y, Schalck A, Yan Y, et al. Delineating copy number and clonal substructure in human tumors from single-cell transcriptomes. Nat Biotechnol. 2021. 10.1038/s41587-020-00795-2 PMC8122019 33462507

[48] Rodriguez-Meira A, Buck G, Clark SA, Povinelli BJ, Alcolea V, Louka E (2019). Unravelling intratumoral heterogeneity through high-sensitivity single-cell mutational analysis and parallel RNA sequencing. Mol Cell.

[49] Maniatis S, Petrescu J, Phatnani H (2021). Spatially resolved transcriptomics and its applications in cancer. Curr Opin Genet Dev.

[50] Csillery K, Blum MG, Gaggiotti OE, Francois O (2010). Approximate Bayesian Computation (ABC) in practice. Trends Ecol Evol.

[51] Jiang B, Wu T-y, Zheng C, Wong WH (2017). Learning summary statistic for approximate Bayesian computation via deep neural network. Statistica Sinica.

[52] Mondal M, Bertranpetit J, Lao O (2019). Approximate Bayesian computation with deep learning supports a third archaic introgression in Asia and Oceania. Nat Commun.

[53] Niida A, Nagayama S, Miyano S, Mimori K (2018). Understanding intratumor heterogeneity by combining genome analysis and mathematical modeling. Cancer.

